# Effect of Celery Seed (*Apium graveolens* L.) Administration on the Components of Metabolic Syndrome, Insulin Sensitivity, and Insulin Secretion: A Clinical Trial

**DOI:** 10.3390/ph19010110

**Published:** 2026-01-07

**Authors:** Miriam de J. Escobedo-Gutiérrez, Marisol Cortez-Navarrete, Esperanza Martínez-Abundis, Karina G. Pérez-Rubio

**Affiliations:** 1 Institute of Experimental and Clinical Therapeutics, Department of Physiology, Health Science University Center, University of Guadalajara, Sierra Mojada 950, Col. Independencia, Guadalajara 44340, Jalisco, Mexico; miriam.escobedo3107@alumnos.udg.mx (M.d.J.E.-G.); marisol.cortez@academicos.udg.mx (M.C.-N.); 2 Internal Medicine Unit, J. Guadalupe Montenegro 2123B, Col. Obrera Centro, Guadalajara 44140, Jalisco, Mexico; esperanzamartnezabundi@yahoo.com

**Keywords:** celery seed, insulin sensitivity, insulin secretion, metabolic syndrome, nutraceutical

## Abstract

**Background/Objectives**: Metabolic syndrome (MetS) is a group of cardiometabolic risk factors whose current management relies on lifestyle changes and pharmacological interventions, frequently involving multiple medications. Therefore, the demand for therapies capable of delivering comprehensive management of MetS is increasing. In this context, nutraceuticals such as celery seed have attracted increasing scientific interest. This study aimed to evaluate the effect of celery seed (*Apium graveolens* L.) administration on the components of MetS, insulin sensitivity, and insulin secretion. **Methods**: A randomized, double-blind, placebo-controlled clinical trial was carried out in 28 patients with MetS. Fourteen patients randomly received celery seed (150 mg/day) for 12 weeks, and 14 subjects received a placebo. Clinical and laboratory determinations were evaluated at baseline and the end of the study. **Results**: After celery seed administration, patients showed a significant decrease in their systolic blood pressure (SBP) (121.0 ± 9.7 mmHg vs. 115.7 ± 12.8 mmHg, *p* = 0.005), diastolic blood pressure (DBP) (82.2 ± 5.9 mmHg vs. 78.5 ± 8.6 mmHg, *p* = 0.013), triglycerides (TG) (2.3 ± 0.9 mmol/L vs. 1.8 ± 0.6 mmol/L, *p* = 0.016), very low-density lipoprotein (VLDL) (0.4 ± 0.1 mmol/L vs. 0.3 ± 0.1 mmol/L, *p* = 0.016) and uric acid (297.4 ± 53.5 µmol/L vs. 261.7 ± 53.5 µmol/L, *p* = 0.009). Insulin sensitivity and insulin secretion showed no statistically significant differences in the celery seed group. **Conclusions**: Celery seed administration significantly reduced SBP, DBP, TG, VLDL, and uric acid. The protocol was registered at ClinicalTrials.gov with the identifier NCT06061926.

## 1. Introduction

Metabolic syndrome (MetS) is a cluster of cardiometabolic risk factors that include central obesity, impaired glucose metabolism, elevated blood pressure (BP), hypertriglyceridemia, and reduced high-density lipoprotein cholesterol (HDL-c) [1]. MetS is highly prevalent globally, currently impacting 25% of the adult population with epidemiological trends demonstrating a sustained rise in prevalence [2]. According to the International Diabetes Federation (IDF), MetS is diagnosed by the presence of an increased waist circumference (WC) (≥80 cm in women and ≥90 cm in men) in combination with at least two of the following diagnostic criteria: BP (≥130/85 mmHg), fasting plasma glucose (FPG) (≥5.6 mmol/L), triglycerides (TG) (≥1.7 mmol/L), or reduced HDL-c (<1.03 mmol/L in men and <1.29 mmol/L in women) [3].

The first line of treatment of MetS is a change in lifestyle (weight loss, dietary habits, physical activity and sleep hygiene) [4]. However, given the difficulty of sustaining lifestyle modifications over time, pharmacological approaches are frequently required [4]. From this perspective, nutraceuticals refer to foods or food-derived components that combine nutritional and pharmacological properties, providing health benefits that may include the prevention or treatment of diseases. These compounds are typically administered in pharmaceutical forms and possess clinically demonstrated efficacy [5]. Among them, celery seed extract has been reported to exert beneficial effects on several components of MetS [6,7,8,9,10].

Celery (*Apium graveolens* L.) is a biennial plant that belongs to order *Apiales* and family *Apiaceae* [11,12]. Originally from southern of Europe, celery is cultivated in the United States of America, France, Germany, United Kingdom, Italy, Hungary, Belgium and Netherlands [12,13]. Celery seeds contain multiple bioactive compounds, including flavonoids (luteolin and apigenin), essential oils (d-limonene and selinene), and phthalides such as 3-n-butylphthalide (NBP), sedanonic anhydride, and sedanenolide [10,14].

Preclinical research in animal models of metabolic disease [15,16,17,18,19], as well as emerging clinical evidence [6,7,8,20,21], suggest that celery seed supplementation may improve several components of MetS. Nevertheless, human data remains limited and heterogeneous, with considerable variability in study populations, outcome measures, and the type of celery interventions evaluated. Consequently, randomized, double-blind, placebo-controlled trials evaluating standardized celery seed extracts in individuals with MetS, while simultaneously assessing multiple MetS components together with validated indices of insulin sensitivity and insulin secretion, have not been conducted to date. Therefore, the present study aimed to evaluate the effects of celery seed (*Apium graveolens* L.) administration on the components of Mets, insulin sensitivity, and insulin secretion.

## 2. Results

### 2.1. Study Population

**Figure 1:**
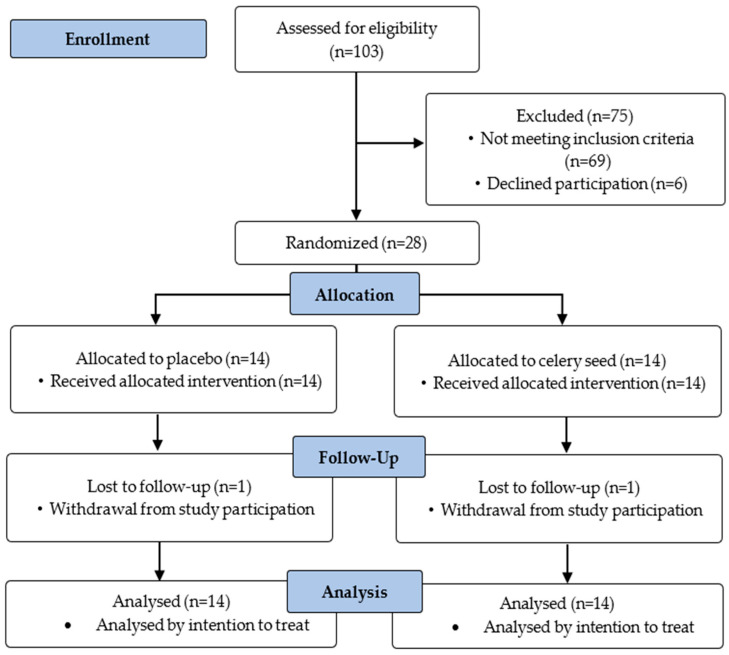
Flow Diagram of Participant Selection.

A total of 103 patients were screened for eligibility. Sixty-nine did not meet the inclusion criteria, and six declined to participate. Consequently, 28 participants were randomized. Of these, 26 completed the 12-week intervention, while one subject from each group (celery seed and placebo) withdrew for reasons unrelated to the intervention. However, all randomized participants (*n* = 28) were included in the final analysis according to the intention-to-treat principle (Figure 1).

The celery seed group included 64% (9/14) women and 36% (5/14) men, while the placebo group consisted of 43% (6/14) women and 57% (8/14) men. The difference in sex distribution between the groups was not statistically significant (*p* = 0.25). The mean ages were similar between groups (47.6 ± 8.6 years in the celery seed vs. 46.0 ± 6.9 years for placebo, *p* = 0.59). Baseline clinical and laboratory characteristics did not differ significantly between groups (Table 1).

### 2.2. Effects of Celery Seed and Placebo on Clinical and Biochemical Parameters

After 12 weeks of intervention, participants receiving celery seed showed significant reductions in systolic blood pressure (SBP) (*p* = 0.005), diastolic blood pressure (DBP) (*p* = 0.013), TG (*p* = 0.016), very low-density lipoprotein (VLDL) (*p* = 0.016), and uric acid (*p* = 0.009) compared with baseline values. Insulin sensitivity and insulin secretion showed no statistically significant differences in the celery seed group. (Table 1).

The placebo group did not show significant changes in any clinical or laboratory variable during the study period (Table 1).

As shown in Table 2, the celery seed group exhibited significantly greater reductions compared with placebo in SBP (*p* = 0.001), DBP (*p* = 0.011), and uric acid (*p* = 0.001) levels. These between-group differences were associated with a large effect size for SBP (*r*= −0.61) and uric acid (*r* = −0.61), and a moderate effect size for DBP (*r* = −0.48), TG *(r* = −0.35) and VLDL *(r* = −0.35). All remaining anthropometric, glycemic, lipid, and insulin-related variables showed small effect size (*r* < −0.20).

To further contextualize the clinical relevance of BP and lipid responses, responder analyses were performed. In the celery seed group, 2/14 participants achieved a reduction in SBP of ≥10 mmHg, whereas none in the placebo group met this criterion. When a lower SBP reduction threshold (≥5 mmHg) was considered, 7/14 participants in the celery seed group achieved this level of reduction, compared with none in the placebo group. Similarly, 7/14 participants in the celery seed group achieved a ≥20% reduction in TG levels, compared with 1/14 participants in the placebo group.

Additional exploratory subgroup analyses were conducted to explore the consistency of treatment effects across selected baseline characteristics. Within the celery seed group, when stratified by sex (women, *n* = 9; men, *n* = 5), significant reductions in SBP and DBP were observed among women, whereas men showed significant reductions in TG, low-density lipoprotein cholesterol (LDL-c), and VLDL concentrations. No sex-specific changes were observed for uric acid.

Subgroup analyses based on baseline BP status were performed using IDF criteria for MetS. Although none of the participants had established hypertension at baseline, 2/14 participants in the celery seed group presented elevated SBP (≥130 mmHg) and 6/14 presented elevated DBP (≥85 mmHg). Significant reductions in both SBP and DBP were observed among participants with normal baseline BP values, whereas no statistically significant changes were detected among those with elevated baseline BP.

Finally, stratification by baseline TG levels revealed that within the celery seed group, 10/14 participants presented hypertriglyceridemia at baseline, whereas 4/14 had normal values. A statistically significant reduction in TG levels was observed among participants with elevated baseline TG, while no significant changes were detected in those with normal baseline levels.

Across all subgroup analyses, no statistically significant changes were observed within the placebo group.

Given the small overall sample size and the unequal distribution of participants across subgroups, these subgroup analyses should be interpreted with caution and are intended to be hypothesis-generating rather than confirmatory.

### 2.3. Adverse Events and Safety Profile

No significant differences were found between groups regarding the incidence of adverse effects, and no serious adverse events occurred during the study. In the celery seed group, the most frequently reported adverse events were mild diarrhea (7.1%) and abdominal distension (7.1%). In the placebo group, headache (21.4%) was the most common, followed by nausea (7.1%), diarrhea (7.1%), abdominal distension (7.1%), and constipation (7.1%). Celery seed was well tolerated. All reported adverse events were mild, transient, and resolved spontaneously without requiring discontinuation of the intervention. These findings support a favorable short-term safety profile of the intervention at the evaluated dose.

## 3. Discussion

Although several pharmacological therapies are currently available for managing MetS, no single medication has been approved to comprehensively address all of its components. Therefore, identifying safe, accessible, and effective therapeutic alternatives remains a priority. To the best of our knowledge, this clinical trial is the first to evaluate the effect of celery seed on the components of MetS, insulin sensitivity, and insulin secretion among individuals diagnosed with MetS. To better contextualize the novelty and clinical scope of our findings, a systematic comparison between the present study and other related clinical trials evaluating celery-based interventions is summarized in Table 3.

The present study makes several unique scientific and clinical contributions to the field of nutraceutical research in metabolic disorders. First, it represents the first clinical evaluation of a standardized, NBP-rich celery seed extract in individuals with MetS, a population characterized by early cardiometabolic dysfunction rather than established disease. Second, this is the first celery seed intervention study to incorporate validated dynamic indices of insulin sensitivity and insulin secretion, including the Matsuda and Stumvoll indices, which are strongly correlated with the hyperinsulinemic–euglycemic clamp. Third, unlike prior clinical studies [6,7,8,20,22], which have primarily focused on individual outcomes such as BP, lipid profile, or glycemic parameters, the present trial contributes to the existing literature by concurrently evaluating multiple components of MetS alongside validated indices of insulin sensitivity and insulin secretion, within a double-blind, placebo-controlled design using a standardized celery seed extract. Finally, by targeting individuals with MetS, a population characterized by early cardiometabolic dysfunction and increased risk of progression to T2DM, our findings support the potential role of nutraceutical interventions as preventive strategies aimed at mitigating cardiometabolic risk before pharmacological therapy is required.

In our study, patients who received celery seed showed significant reductions in SBP, DBP, TG, VLDL, and uric acid levels.

An important component of MetS is high BP. Increased visceral adipose tissue can increase angiotensin II (Ang II) levels [1], which contributes to vascular dysfunction by promoting the generation of reactive oxygen species and reducing nitric oxide synthesis. Moreover, Ang II impairs the vasodilatory action of insulin, resulting in vasoconstriction and a subsequent rise in BP [23].

Clinically, even modest reductions in BP are meaningful; a 10 mmHg decrease in SBP has been associated with a lower risk of coronary artery disease, stroke, and heart failure, while a 5 mmHg decrease can reduce cardiovascular events by approximately 10% [24].

In our trial, celery seed significantly decreased SBP and DBP. These findings are consistent with previous studies. Madhavi et al. reported that 150 mg/day of celery seed extract standardized to 85% NBP for six weeks significantly decreased SBP and DBP in hypertensive patients [7]. Similarly, Gharouni et al. observed a significant decrease in SBP and DBP following administration of 6 g/day of celery seed for two weeks in patients with hypertension [6]. The antihypertensive activity of celery seed may derive from NBP and apigenin, which can exert calcium channel-blocking and vasodilatory effects via nitric oxide synthase activation. These mechanisms support its BP-lowering potential [10,17].

Hypertriglyceridemia is another key component of MetS, which is a consequence of insulin resistance (IR) in MetS. IR promotes enhanced lipolysis, resulting in elevated levels of free fatty acids (FFA), which in turn stimulate the hepatic synthesis of TG and VLDL [1]. In this study, celery seed administration led to a significant reduction in TG levels and a parallel decrease in VLDL concentrations was also observed. Our findings corroborate the outcomes of the clinical trial conducted by Shayani et al., where a significant reduction in TG after the administration of 1.34 g/day of celery seed for 4 weeks was found [8]. In experimental studies, similar results have been reported after the administration of celery seed extract in mouse models of induced obesity or diabetes [18,19,25].

Although the precise mechanism underlying the TG-lowering effect of celery seed has not been fully elucidated, evidence suggests it involves peroxisome proliferator-activated receptor-α agonist (PPAR-α) activity, which induces transactivation and upregulation of target genes such as the lipoprotein lipase (LPL) gene, leading to increased LPL activity and enhanced TG hydrolysis. Furthermore, the suppression of hepatic lipogenesis and reduced intestinal lipid absorption have been proposed as additional contributing mechanisms [10,26].

In our study, celery seed reduced TG concentrations by approximately 19%, a magnitude comparable to that typically achieved with fibrates or statins in patients with mixed dyslipidemia [27,28]. Lowering TG is clinically relevant, as even moderate reductions are associated with meaningful cardiovascular benefits, particularly in individuals with hypertriglyceridemia [29]. 

Hyperuricemia has been correlated with several diseases such as gout, MetS, hypertension, diabetes, kidney disease, and cardiovascular disease. This pro-oxidant metabolite can induce inflammation and endothelial dysfunction [30]. In the present study, a significant reduction in uric acid concentrations was observed. This finding is consistent with a prior preclinical study where hyperuricemia was experimentally induced using potassium oxonate [31]. The proposed mechanism likely involves the inhibition of xanthine oxidase, the enzyme responsible for catalyzing the conversion of hypoxanthine to xanthine, as well as xanthine dehydrogenase, which facilitates the subsequent transformation of xanthine into uric acid [31].

Visceral obesity is a key determinant of Mets [1]. This type of obesity is associated with lipotoxicity and cellular dysfunction, particularly in the liver, pancreas and skeletal muscle, thereby contributing to the progression of the other components of MetS [23]. WC is an easy method to standardize and use in clinical practice. It can be used to assess adiposity in the abdominal area. It is also strongly associated with cardiovascular diseases and mortality; consequently, its reduction contributes to decreased cardiometabolic risk [32].

No significant changes were observed in WC in our study, consistent with earlier clinical evidence. Mohsenpour et al. reported no statistically significant changes in WC after 12 weeks of administering 750 mg/day of celery powder (prepared from fresh celery, including stems and leaves) to patients with type 2 diabetes mellitus (T2DM) [22]. Although preclinical data [15] have suggested anti-obesity effects of celery’s flavonoid component, luteolin, our study’s short intervention period and absence of a controlled diet or structured physical activity program may have attenuated these effects. Future studies incorporating lifestyle monitoring and longer interventions are needed to clarify celery seed’s impact on abdominal adiposity.

In the context of IR, lipolysis and FFA levels increase, which lead to an enhanced synthesis of TG and VLDL [1]. In addition, synthesis of LPL, essential for removing TG-rich lipoproteins, is diminished [23]. Together, these mechanisms lead to reduced HDL-c levels [1,23]. Robust evidence demonstrates that reduced HDL-c levels are linked to an increased risk of cardiovascular disease [33].

In this study, HDL-c concentrations remained stable, no statistically significant differences were observed in women or men. Similar findings were reported by Mohammad et al., in patients with T2DM; no significant changes in HDL-c levels were observed [22]. In contrast, Shayani et al. documented a significant increase in HDL-c in hypertensive patients [8]. This effect may be related to an enhancement of lecithin–cholesterol acyltransferase activity, a mechanism proposed to contribute to HDL-c elevation with celery seed [10,34]. Discrepancies among trials may be related to differences in dosage, extract composition, and participant characteristics.

A further component of MetS is hyperglycemia, which is closely related to IR. In this state, impaired insulin signaling promotes increased hepatic gluconeogenesis, reduces glucose uptake in skeletal muscle, and diminishes muscle glycogen synthesis [10,23]. The glucose-lowering effect of celery seed and some of its bioactive components has been confirmed in several preclinical trials using mouse models of induced diabetes and models of obesity caused by a high-fat diet [11,16,19,25,35]. Several clinical trials have reported significant reductions in FPG, including the study by Yusni et al. in patients with prediabetes. Patients were given 250 mg/3 times per day of celery leaf for 12 days and after the intervention period, glucose levels were significantly reduced [20].

In our study, FPG in the celery seed group showed a modest decline that did not reach statistical significance. Although previous randomized trials [8,20] and meta-analysis [21] have reported modest improvements in glycemic parameters, several factors may explain the absence of significant effects in the present study. Participants exhibited relatively modest baseline glucose levels, which may have limited the potential for detecting significant changes. In addition, differences in study populations, extract formulations, dosage, and intervention duration across published trials may contribute to heterogeneous glycemic responses.

Insulin sensitivity and first-phase insulin secretion were assessed using the Matsuda and Stumvoll indices, respectively. The Matsuda index shows a correlation of 0.73 [36,37] with the gold standard hyperinsulinemic-euglycemic clamp, whereas the Stumvoll index correlates at 0.78 [38,39]. Total insulin secretion (AUC insulin/AUC glucose) was also evaluated. In this study, no significant differences were observed in the treatment group for the Matsuda index and Stumvoll index, or total insulin secretion. These results are consistent with those reported by Mohsenpour et al., in patients with T2DM who found no statistically significant differences on insulin concentrations within the intervention group [22].

The absence of significant changes in these indices may be explained by the characteristics of the study population and the nature of these dynamic measures. Both the Matsuda and Stumvoll indices are strongly influenced by baseline IR and β-cell dysfunction, which were relatively mild in the present cohort. In addition, celery seed supplementation is not known to exert direct insulinotropic effects, and no significant changes in body weight or central adiposity were observed factors commonly associated with improvements in insulin sensitivity.

However, a preclinical study has identified a possible mechanism through which a bioactive compound from celery seed, luteolin, may enhance insulin sensitivity and secretion. Luteolin has been shown to facilitate the translocation of glucose transporter type 4 to the plasma membrane, thereby improving glucose uptake in adipose tissue [40]. In addition, celery seed exhibits antioxidant properties (by inhibiting lipid peroxidation) and anti-inflammatory effects (by reducing interleukin-6 and tumor necrosis factor-alpha levels), which may contribute to the repair and proliferation of pancreatic β-cells and the enhancement of insulin secretion [11,16]. These mechanistic observations highlight the need for further clinical trials.

From a clinical perspective, the observed reductions in SBP, DBP, TG, VLDL, and uric acid are of particular relevance in individuals with MetS, in whom the simultaneous presence of multiple cardiometabolic risk factors substantially increases cardiovascular risk [41]. Even modest improvements across several components, rather than large effects on a single parameter, may translate into meaningful risk reduction when applied at early stages of cardiometabolic dysfunction [42].

The use of a standardized, NBP-rich celery seed extract has practical implications for nutraceutical development, as standardization ensures reproducibility, quality control, and facilitates more reliable clinical translation compared with non-standardized preparations. The favorable safety and tolerability profile observed in this trial further supports the feasibility of incorporating celery seed–based nutraceuticals as adjunctive strategies for cardiometabolic risk management.

From a public health perspective, nutraceutical interventions targeting individuals with MetS may represent accessible and practical strategies to complement lifestyle modifications and potentially delay the initiation of pharmacological therapy in populations at elevated cardiometabolic risk.

Although the present study provides clinically relevant evidence, further mechanistic studies are warranted to elucidate the biological pathways underlying the observed effects of celery seed supplementation. Future studies should explore the impact of standardized NBP-rich extracts on vascular function, including endothelial nitric oxide bioavailability, renin–angiotensin system activity, and calcium channel modulation, which may contribute to BP regulation. In addition, mechanistic studies focusing on lipid metabolism are needed to clarify the role of pathways such as PPAR-α activation, LPL regulation, and hepatic lipogenesis in mediating TG-lowering effects. Incorporating biomarkers of oxidative stress, inflammation, and insulin signaling will be essential to understand and guide future clinical applications.

The strengths of this study include its randomized, double-blind, placebo-controlled design and the use of a commercially available celery seed extract standardized to 85% of NBP. To the best of our knowledge, this is the first trial in patients with MetS to simultaneously evaluate all clinical components of the syndrome alongside insulin sensitivity and secretion. These metabolic parameters were assessed using validated indices, such as the Matsuda and Stumvoll indices, which demonstrate strong correlation with the hyperinsulinemic-euglycemic clamp—the gold standard in the field. Furthermore, the intervention was characterized by excellent tolerability, high adherence, and a notably low attrition rate.

Several limitations of this study should be acknowledged. First, the relatively small sample size and the short intervention period may limit statistical power and the generalizability of the findings, and the results should therefore be interpreted with caution and confirmed in larger, more diverse populations. Second, although the study demonstrated clinically relevant cardiometabolic effects, the underlying mechanisms of action were not directly assessed, which limits the ability to confirm the biological pathways responsible for the observed outcomes. Third, lifestyle-related factors known to influence cardiometabolic outcomes, including dietary intake, habitual physical activity, sleep patterns, and stress levels, were not systematically monitored or quantitatively assessed and may have contributed to interindividual variability in treatment response. Finally, menopausal status was not systematically evaluated or used for stratification, which may have influenced metabolic outcomes given the known effects of hormonal status on cardiometabolic risk.

These limitations highlight the need for future clinical trials with larger sample sizes, longer intervention and follow-up periods, more rigorous control of confounding factors, and the evaluation of varied dosing regimen and different celery seed preparations to confirm these findings and to better define the potential therapeutic role of celery seed in individuals with MetS.

## 4. Materials and Methods

### 4.1. Study Design

A randomized, double-blind, placebo-controlled clinical trial was conducted in 28 patients with MetS to evaluate the effect of celery seed administration on the components of MetS insulin sensitivity and insulin secretion.

### 4.2. Selection Criteria

Subjects were eligible if they met IDF criteria for MetS. Inclusion criteria included adults (30–60 years), IMC between 25.0 and 34.9 kg/m^2^, and stable BW (without variations above or under 5%) for at least 3 months prior to enrollment. None of the subjects were receiving pharmacological treatment for MetS. Additionally, participants were required to have FPG ˂ 7.0 mmol/L, c-LDL < 4.0 mmol/L, TG < 5.6 mmol/L, SBP < 140 mmHg, and DBP < 90 mmHg.

Patients did not engage in heavy physical activity or were extremely sedentary. All individuals were nonsmokers, lived in the same geographical area, and shared a similar socioeconomic background. Women of reproductive age were required to use a contraception method.

Exclusion criteria included confirmed or suspected pregnancy, breastfeeding, difficulty swallowing capsules, or the presence of uncontrolled renal, hepatic, cardiovascular, or thyroid disorders.

### 4.3. Randomization

Simple randomization was performed by an independent investigator using a random number list computer-generated sequence. A total of 28 patients were randomly allocated using a numerical code to one of the two treatment groups. To ensure blinding, the celery seed extract and placebo capsules were identical in size, appearance, and packaging. Both interventions were labeled with coded identifiers by an independent researcher not involved in participant enrollment, follow-up, or data analysis. Both participants and investigators were blinded to treatment allocation, which was disclosed only after data collection and analysis were completed.

### 4.4. Pharmacological Intervention

Fourteen participants received 150 mg/day (75 mg oral capsules twice daily, before breakfast and dinner) of a commercially available celery seed extract (Natural Factors^®^, Celery Seed Standardized Extract, Coquitlam, BC, Canada; Lot 5018422) for 12 weeks, while the remaining 14 patients received a placebo (calcined magnesia) in an identical form and dosage. The celery seed dose was selected based on prior research conducted by Madhavi et al. [7], who used celery seed standardized to 85% NBP and reported beneficial effects on BP. The product used in this study consists of a standardized extract of *Apium graveolens* seed, with NBP (85%) as the declared bioactive compound. All participants received capsules from the same production batch. The product was manufactured under Good Manufacturing Practice standards applicable to dietary supplements.

All participants received general recommendations on nutrition and physical activity, which were reinforced during follow-up visits. Treatment adherence was assessed by pill counts and review of participants’ personal diaries, with adherence defined as consumption of ≥80% of prescribed doses. Participants were instructed to record any drug-related adverse events in their diaries. The occurrence of adverse events was also assessed at each visit through personal interviews, and all findings were reported to the Ethics Committee.

### 4.5. Clinical and Laboratory Determinations

Participants attended five scheduled visits during the study period. Height was measured with participants standing barefoot and with their heads positioned according to the Frankfort horizontal plane. BW and body fat percentage were obtained using a bioimpedance analyzer (InBody^®^ 270, Seoul, Republic of Korea). BMI was calculated as weight (kg) divided by height squared (m^2^). WC was measured with a flexible tape (Lufkin^®^ W606PM, Sparks, MD, USA) at the midpoint between the iliac crest and the lowest rib along the midaxillary line. All anthropometric assessments were conducted with participants wearing light clothing, without shoes, socks or topical cream and after bladder emptying. BP was measured using a digital sphygmomanometer (OMRON^®^, model HEM-7130, Kyoto, Japan) after a 15-min rest in a seated position. The average of three consecutive systolic and diastolic readings was recorded.

Blood samples were collected at 8:00 a.m. after an 8–12 h overnight fast. For female participants, sample collection was conducted during the follicular phase (days 3–8) of the menstrual cycle. Samples were centrifuged at 851× *g* (2500 rpm) [Beckman Coulter; Allegra^TM^ X-22 R^®^ centrifuge, Brea, CA, USA]. Serum was aliquoted and immediately analyzed for fasting FPG, total cholesterol (TC), TG, HDL-c, alanine aminotransferase, aspartate aminotransferase creatinine and uric acid. These parameters were measured using colorimetric methods with an automated analyzer (Erba XL-100^®^, Mannheim, Germany), with intra- and interassay coefficients of variation < 1% and 2%, respectively. LDL-c was calculated using the Friedewald formula adjusted for mmol/L: (LDL-c = TC − HDL-c − [TG/2.2]), and VLDL was estimated by the ratio TG/2.2.

Also, a 2-h oral glucose tolerance test was performed using a standard 75 g glucose load. Blood samples were collected at baseline and at 30, 60, 90, and 120 min to measure plasma glucose and serum insulin levels. These determinations were used to calculate indices of insulin sensitivity and secretion. Plasma glucose concentrations were measured using an enzymatic colorimetric method following the manufacturer’s instructions, with routine calibration performed according to standard laboratory procedures. Serum insulin concentrations were evaluated using a commercially available enzyme-linked immunosorbent assay (ELISA) kit (IN374S; Calbiotech, Inc., El Cajon, CA, USA), validated for clinical use, with intra- and inter-assay coefficients of variation below 5%. Internal quality control procedures were applied for both glucose and insulin measurements, and all analyses were performed in the same certified laboratory.

Insulin sensitivity was calculated with Matsuda index (10,000/√ (glucose_0′_ × insulin_0′_) (glucose_mean 0′–120′_ × insulin_mean 0′–120′_) [36]. The first phase insulin secretion was determined with the Stumvoll index (1283 + 1.829 × insulin_30′_ − 138.7 × glucose_30’_ + 3.772 × insulin_0′_) [38]. Total insulin secretion was calculated through the ratio of the AUC of insulin from 0 to 120 min (AUC insulin/AUC glucose) [39].

### 4.6. Statistical Analysis

Sample size was estimated using a clinical trial formula with a 95% confidence level and 80% statistical power [43]. Calculations were performed for each primary outcome, and the largest sample size obtained—corresponding to insulin secretion—was selected to ensure adequate power across all variables. The analysis assumed a standard deviation (SD) for insulin secretion of 0.16 and an expected between-group difference of at least 0.20, obtaining a total of 14 patients per group, including 20% of expected loss [44].

Data were expressed as mean ± SD and analyzed using the International System of Units. Intra and intergroup differences were tested by the Wilcoxon signed-rank and Mann–Whitney U tests, respectively. Intergroup difference analyses were performed using within-group changes (Δ from baseline to week 12), and effect sizes were calculated using the effect size *r* (*r* = Z/√N) to estimate the magnitude of between-group differences for the main efficacy outcomes. Also, an intention-to-treat analysis was conducted. A *p*-value of ≤0.05 was considered statistically significant. Statistical analyses were conducted using SPSS version 25.

### 4.7. Ethical Considerations

The study protocol was approved by the local Ethics Committee, and all participants provided written informed consent prior to enrollment. The protocol was registered at ClinicalTrials.gov with the identifier NCT06061926.

## 5. Conclusions

Celery seed administration significantly reduced SBP, DBP, TG, VLDL, and uric acid levels. These results suggest that celery seed may act as an adjuvant therapy for patients with MetS. Nevertheless, long-term studies with larger cohorts are warranted to confirm and expand upon these findings.

## Figures and Tables

**Table 1:** Characteristics before and after intervention (Mean ± SD).

	Placebo Group						Celery Seed Group					
	Baseline			12 weeks			Baseline			12 weeks		
	*n* = 14			*n* = 14			*n* = 14			*n* = 14		
Body weight, kg	88.1	±	12.0	88.5	±	12.1	81.2	±	10.2	80.9	±	11.3
BMI, kg/m^2^	31.5	±	3.1	31.6	±	3.2	30.9	±	2.7	30.7	±	3.0
WC women, cm	105.2	±	5.1	105.4	±	5.6	100.8	±	4.7	100.5	±	5.6
WC men, cm	106.6	±	11.6	107.0	±	11.7	103.9	±	8.5	104.3	±	9.6
Body fat, %	37.1	±	10.0	37.4	±	9.6	39.1	±	7.0	39.2	±	7.6
SBP, mmHg	122.2	±	13.1	125.4	±	9.6	121.1	±	9.7	115.7	±	12.8 **
DBP, mmHg	82.0	±	6.5	82.3	±	5.4	82.2	±	5.9	78.5	±	8.6 **
FPG, mmol/L	6.0	±	0.3	5.9	±	0.4	5.9	±	0.3	5.6	±	0.5
TG, mmol/L	2.2	±	1.2	2.1	±	1.1	2.3	±	0.9	1.8	±	0.6 **
TC, mmol/L	4.7	±	0.9	4.8	±	0.9	4.4	±	0.5	4.4	±	0.7
LDL-c, mmol/L	2.6	±	0.8	2.8	±	0.7	2.3	±	0.7	2.5	±	0.8
VLDL, mmol/L	0.4	±	0.2	0.4	±	0.2	0.4	±	0.1	0.3	±	0.1 **
HDL-c women, mmol/L	1.1	±	0.2	1.0	±	0.2	1.0	±	0.2	1.0	±	0.2
HDL-c men, mmol/L	1.0	±	0.1	1.1	±	0.1	0.9	±	0.0	1.0	±	0.1
Creatinine, µmol/L	70.7	±	8.8	70.7	±	0.1	61.8	±	8.8	61.8	±	8.8
Uric acid, µmol/L	291.4	±	65.4	309.3	±	83.2	297.4	±	53.5	261.7	±	53.5 **
AST, U/L	24.6	±	11.4	23.6	±	11.6	24.6	±	18.6	25.0	±	10.8
ALT, U/L	34.7	±	12.5	32.9	±	13.0	31.5	±	22.7	33.8	±	24.4
Matsuda index	2.1	±	1.2	2.0	±	0.9	2.3	±	0.9	2.3	±	1.6
Stumvoll index	2262.1	±	1105.4	2299.5	±	1021.6	1532.0	±	720.9	1645.7	±	650.3
Total insulin secretion	0.7	±	0.3	0.8	±	0.3	0.5	±	0.2	0.5	±	0.2

** *p* < 0.01; between baseline and 12-week intragroup measurement (Wilcoxon rank test). BMI: body mass index, WC: waist circumference, SBP: systolic blood pressure, DBP: diastolic blood pressure, FPG: fasting plasma glucose, TG: triglycerides, TC: total cholesterol, LDL-c: low-density lipoprotein cholesterol, VLDL: very low-density lipoprotein, HDL-c: high-density lipoprotein cholesterol, AST: aspartate aminotransferase, ALT: alanine aminotransferase.

**Table 2:** Change values from baseline between groups.

	Placebo Group			Celery Seed Group			*p* *	*R* **
	*n* = 14			*n* = 14				
Body weight, kg	0.37	±	1.41	−0.34	±	3.12	0.419	−0.15
BMI, kg/m^2^	0.13	±	0.52	−0.16	±	1.22	0.489	−0.13
WC women, cm	0.25	±	1.44	−0.29	±	2.99	1.000	0.00
WC men, cm	0.39	±	0.87	0.36	±	2.20	0.366	−0.17
Body fat, %	0.35	±	1.48	−0.13	±	1.71	0.852	−0.04
SBP, mmHg	3.14	±	7.81	−5.43	±	5.21	0.001	−0.61
DBP, mmHg	0.57	±	2.95	−3.72	±	4.79	0.011	−0.48
FPG, mmol/L	−0.11	±	0.42	−0.31	±	0.57	0.490	−0.13
TG, mmol/L	−0.06	±	0.39	−0.45	±	0.61	0.066	−0.35
TC, mmol/L	0.13	±	0.68	0.03	±	0.65	0.713	−0.07
LDL-c, mmol/L	0.16	±	0.63	0.23	±	0.70	0.854	−0.03
VLDL, mmol/L	−0.01	±	0.07	−0.09	±	0.12	0.066	−0.35
HDL-c women, mmol/L	−0.03	±	0.08	−0.01	±	0.14	0.376	−0.17
HDL-c men, mmol/L	0.02	±	0.18	0.02	±	0.04	0.266	−0.21
Creatinine, µmol/L	−1.23	±	6.70	−1.67	±	5.26	0.982	0.00
Uric acid, µmol/L	14.45	±	35.60	−34.56	±	29.70	0.001	−0.61
AST, U/L	−1.04	±	9.00	0.38	±	16.38	0.357	−0.17
ALT, U/L	−1.81	±	11.92	2.32	±	17.68	0.963	−0.01
Matsuda index	−0.13	±	0.54	−0.26	±	1.38	0.581	−0.10
Stumvoll index	37.40	±	315.05	113.64	±	397.45	0.323	−0.19
Total insulin secretion	0.02	±	0.10	0.03	±	0.11	0.696	−0.07

* Statical analysis: Mann–Whitney U test. ** Effect size (*r*) was calculated from the Z value of the Mann–Whitney U test. BMI: body mass index, WC: waist circumference, SBP: systolic blood pressure, DBP: diastolic blood pressure, FPG: fasting plasma glucose, TG: triglycerides, TC: total cholesterol, LDL-c: low-density lipoprotein cholesterol, VLDL: very low-density lipoprotein, HDL-c: high-density lipoprotein cholesterol, AST: aspartate aminotransferase, ALT: alanine aminotransferase.

**Table 3:** Systematic comparison of clinical trials evaluating celery-based interventions on cardiometabolic outcomes.

Characteristic	Present Study	Mohsenpour et al. [22]	Shayani Rad et al. [8]	Yusni et al. [20]	Madhavi et al. [7]	Gharouni & Sarkarati [6]
Study design	Randomized, double-blind, placebo-controlled clinical trial	Randomized, double-blind, placebo-controlled pilot trial	Randomized, triple-blind, placebo-controlled, cross-over trial	Quasi-experimental, with control group	Single-arm, open-label pilot study	Experimental (single group)
Population	Patients with MetS	Overweight/obese with T2DM patients	Hypertensive patients	Pre-diabetic participants	Hypertensive patients	Hypertensive patients
Sample size	28 subjects	50 subjects	52 subjects	16 subjects	30 subjects	37 subjects
Intervention	Standardized celery seed extract (Natural Factors^®^) 85% 3-n-butylphthalide	Celery powder (obtained from stem and leaves) Not standardized.	Celery seed extract	Celery leaf extract. Not standardized	Standardized celery seed extract 85% 3-n-butylphthalide	Celery seed powder Not standardized
Daily dose	150 mg/day (75 mg twice daily)	750 mg	1.34 g/day	750 mg/day (250 mg, 3 times a day)	150 mg/day (75 mg twice daily).	6 g/day
Intervention duration	12 weeks	12 weeks	4 weeks	12 days	6 weeks	Not clearly reported (before–after comparison)
Results	↓ SBP ↓ DBP ↓ TG ↓ VLDL ↓ UA ↔ HDL-c ↔ LDL-c ↔ TC ↔ FPG ↔ Matsuda index ↔ Stumvoll index ↔ BW ↔ BMI ↔ WC ↔ BF ↔ AST ↔ ALT	↓ BW ↓ BMI ↓ HC ↑ SBP ↑ DBP ↔ WC ↔ BF ↔ FPG ↔ Insulin ↔ HOMA-IR ↔ HOMA-B ↔ TG ↔ TC ↔ LDL-c ↔ HDL-c ↔ AST ↔ ALT	↓ SBP ↓ DBP ↓ TG ↓ TC ↓ LDL-c ↑ HDL-c ↓ FPG	↓ FPG ↓ PPG ↔ Insulin	↓ SBP ↓ DBP ↔ TC ↔ LDL-c ↔ HDL-c ↔ VLDL	↓ SBP ↓ DBP
Insulin sensitivity/secretion assessment	Matsuda index Stumvoll index	HOMA-IR HOMA-B	Not evaluated	Plasma insulin measured	Not evaluated	Not evaluated

MetS: metabolic syndrome; T2DM: type 2 diabetes mellitus; SBP: systolic blood pressure; DBP: diastolic blood pressure; TG: triglycerides; VLDL: very low-density lipoprotein; UA: uric acid; HDL-c: high-density lipoprotein cholesterol; BW: body weight; BMI: body mass index; WC: waist circumference; BF: body fat; AST: aspartate aminotransferase; ALT: alanine aminotransferase; FPG: fasting plasma; HOMA-IR: homeostatic assessment model for insulin resistance; HOMA-B: homeostatic assessment model for β-cell function; TG: triglycerides; TC: total cholesterol; LDL-c: low-density lipoprotein cholesterol; PPG: postprandial plasma glucose. Parameter changes: ↓: significant decrease; ↑: significant increase; ↔: unchanged. To our knowledge, the present study is the first to simultaneously evaluate a standardized celery seed extract in individuals with MetS and to incorporate validated indices of insulin sensitivity and insulin secretion.

## Data Availability

The original contributions presented in this study are included in the article. Further inquiries can be directed to the corresponding author.

## Abbreviations

MetS	Metabolic syndrome
SBP	Systolic blood pressure
DBP	Diastolic blood pressure
TG	Triglycerides
VLDL	Very low-density lipoprotein
LDL-c	Low-density lipoprotein cholesterol
BP	Blood pressure
HDL-c	High-density lipoprotein cholesterol
IDF	International Diabetes Federation
WC	Waist circumference
FPG	Fasting plasma glucose
NBP	3-n-butylphthalide
Ang II	Angiotensin II
IR	Insulin resistance
PPAR-α	Peroxisome proliferator-activated receptor-α agonist
LPL	Lipoprotein lipase
T2DM	Type 2 diabetes mellitus
FFA	Free fatty acids
TC	Total cholesterol
